# Stage-Specific Alternative Polyadenylation During Human Neural Differentiation Revealed by Integrated Long- and Short-Read Sequencing

**DOI:** 10.3390/biology15010024

**Published:** 2025-12-23

**Authors:** Zheqi Lou, Xianyan Zeng, Tinghui Jiang, Peizhen Du, Jiyao Rao, Xinyan Dai, Haishuang Lin, Yong Zhu

**Affiliations:** 1 College of Basic Medicine, Chongqing Medical University, Chongqing 400016, China; 2 College of Pharmacy, Chongqing Medical University, Chongqing 400016, China; 3 School of Biomedical Engineering, Division of Life Sciences and Medicine, University of Science and Technology of China, Hefei 230000, China; 4 Suzhou Institute for Advanced Research, University of Science and Technology of China, Suzhou 215000, China

**Keywords:** human embryonic stem cells, neural differentiation, alternative polyadenylation, intronic alternative polyadenylation, exon alternative polyadenylation, Oxford Nanopore Technologies, short-read sequencing

## Abstract

Human embryonic stem cell (hESC) neural differentiation involves extensive APA; however, reliance on short-read sequencing in prior studies has offered only a limited view of its complexity and dynamic regulation. Here, we integrated Oxford Nanopore (ONT) long-read sequencing with Illumina short-read sequencing to systematically map the APA landscape during early hESC neural differentiation. Our hybrid approach uncovered remarkable transcriptomic complexity, identifying 20,823 novel transcripts and 8241 previously unannotated poly(A) sites (PASs). We characterized distinct dynamic patterns of 3′ UTR-APA across differentiation and pinpointed *SOX11* as a key APA-regulated target. Furthermore, we observed stage-specific enrichment of intronic APA in NPCs, as exemplified by *SLC1A3*, and performed a comprehensive, large-scale identification of high-confidence exon APA events. These results substantially expand the catalog of PASs during human neural differentiation and provide new insights into how APA-mediated post-transcriptional regulation contributes to cell-fate decisions.

## Simple Summary

The differentiation of pluripotent embryonic stem cells into specialized neural cells is a highly complex and precise process. Alternative polyadenylation (APA), a crucial post-transcriptional regulatory mechanism, plays a significant role in shaping gene expression. In this study, we integrated long- and short-read sequencing to profile the dynamic landscape of APA during early neural differentiation. We identified four distinct dynamic patterns of mRNA 3′ untranslated region (3′ UTR) length variation and uncovered widespread intronic polyadenylation events, particularly during the neural precursor cell (NPC) stage. Among these, *SOX11* and *SLC1A3* were highlighted as novel APA-regulated transcripts. By providing a high-resolution atlas of APA regulation, this work offers new insights into the mechanisms of neural lineage differentiation and supplies valuable molecular clues for exploring the etiology and potential therapeutic avenues of nervous system disorders.

## 1. Introduction

Alternative polyadenylation serves as a pivotal post-transcriptional regulatory mechanism in eukaryotes. In mammals, more than 79% of genes possess multiple PASs. The selection of different PASs during pre-mRNA maturation generates distinct mRNA isoforms, substantially expanding transcriptomic and proteomic diversity [1,2,3]. Based on genomic location, APA is mainly categorized into two types: 3′ UTR-APA, which occurs within the 3′ untranslated region and modulates mRNA stability, translation efficiency, and localization through changes in 3′ UTR length; and upstream region (UR)-APA, where PASs reside in alternative terminal exons, internal exons, or introns upstream of the 3′ UTR. UR-APA alters the protein-coding sequence, often producing truncated protein variants or introducing premature termination codons that can trigger nonsense-mediated mRNA decay (NMD) [4,5,6,7,8].

APA patterns are both tissue-specific and cell state-specific, undergoing global reprogramming during cell fate determination and development, especially in neurodevelopment. Studies indicate that proliferating or undifferentiated cells tend to use proximal PASs, whereas differentiated cells favor distal PAS usage [9,10,11]. Consistent with this, mRNA 3′ UTRs progressively lengthen via APA during mouse embryonic development [9,12,13,14,15]. Notably, when mature neurons are reprogrammed to a stem cell-like state, their APA profiles shift back toward proximal PAS usage [11,12,16,17]. These observations suggest that PAS selection is dynamically and directionally regulated during neuronal differentiation, representing a key layer of gene regulatory networks that control cellular states. Furthermore, neuronal differentiation is known to be regulated by multiple signaling pathways involving neurotrophic factors such as NGF and BDNF [18,19,20]. Dysregulation of APA in molecules related to differentiation has been linked to neurodevelopmental disorders and subsequent neurological diseases. For example, in Rett syndrome (RTT), the pathogenic gene *MECP2* downregulates target genes via APA [21,22].

However, current knowledge of APA dynamics in neural development relies predominantly on short-read sequencing. Standard RNA-seq lacks precise 3′-end information, and although 3′-end-specific library protocols improve accuracy, their limited gene coverage and short read lengths can lead to ambiguous alignment [23]. In addition, short-read-based APA analysis depends strongly on pre-annotated PAS databases [24], potentially obscuring unannotated or context-specific APA events.

Third-generation sequencing technologies, including ONT and Pacific Biosciences (PacBio), enable direct, real-time sequencing of individual, full-length RNA molecules [25,26]. These platforms accurately resolve complex transcript isoforms arising from alternative splicing and polyadenylation. Additionally, it allows for the direct detection of co-transcriptional RNA modifications, opening avenues to study layered post-transcriptional regulation [27,28,29].

In recent years, hybrid sequencing strategies integrating short- and long-read technologies have been successfully employed to APA studies across diverse biological systems, including Drosophila development, the human visual cortex during puberty, hESCs and neurons [30,31,32]. However, existing studies have mainly focused on broader time points or terminal cell types, leaving the APA landscape during early human neural lineage specification, especially the systematic characterization of intronic and exon APA events, still incomplete.

To address this gap, we applied an integrated long- and short-read sequencing approach to dissect APA regulation during the early neural differentiation transition from hESCs to NPCs. Our study aims to: (1) comprehensively map PASs across all genomic regions and identify novel sites; (2) characterize the dynamics of APA in 3′ UTRs, introns, and exons throughout this continuous differentiation process; (3) identify and preliminarily characterize key APA-regulated genes. Together, this work provides a refined resource and new insights into the post-transcriptional regulation underlying neural fate determination.

## 2. Materials and Methods

### 2.1. hESCs Culture

The H9 human embryonic stem cell line was procured from WiCell Research Institute (catalog no. WB68075). Cells were cultivated in mTeSR1 medium (STEMCELL, Vancouver, BC, Canada) supplemented with 1% penicillin–streptomycin on six-well plates freshly coated with Matrigel (Corning, NY, USA, 354277) at a 1:100 dilution. Cells were cultured at 37 °C in a humidified incubator with 5% CO_2_. Cultures were maintained through daily medium replacement and subcultured every four days using ReLeSR (STEMCELL). Regular quality control assays confirmed the expression of pluripotency markers (OCT4 (sc-5279, Santa Cruz, CA, USA) and NANOG (sc-293121, Santa Cruz, CA, USA)), the capacity for teratoma formation in immunodeficient mice, normal karyotype verified by G-banding technique, and the absence of bacterial and mycoplasma contamination. Cells were routinely tested for mycoplasma contamination prior to key experiments. The animal experiments were carried out following the protocols approved by the University of Chongqing Medical University Animal Care and Use Committee. Cells between passages 30 and 35 were used to minimize experimental variability.

### 2.2. hESC Differentiation into Neural Stem Cells (NSCs)

For neural induction, single-cell suspensions of hESCs were seeded onto Matrigel-coated 6-well plates at a density of 2.0 × 10^6^ cells per well. The cultures were initially maintained overnight in complete mTeSR1 medium supplemented with a Rho-associated protein kinase (ROCK) inhibitor to achieve > 90% confluency. The following day, the medium was switched to a neural induction cocktail composed of Essential 6 (E6) basal medium (Invitrogen, Waltham, MA, USA) containing 100 nM LDN193189 (Selleckchem, Houston, TX, USA, #S2618) and 10 μM SB431542 (Selleckchem, #S1067), without ROCK inhibitor. This induction medium was refreshed daily over a 7-day period. Successful differentiation into NSCs was confirmed on day 8 by immunostaining, which demonstrated that the majority of cells expressed the characteristic NSC markers PAX6 and NESTIN.

### 2.3. hESC Differentiation into NPCs

To initiate neural progenitor cell differentiation, single-cell suspensions of hESCs were plated in Matrigel-coated 6-well plates at a density of 2.0 × 10^6^ cells per well. The cultures were maintained overnight in complete mTeSR1 medium containing a ROCK inhibitor to achieve > 90% confluence. The following day, the medium was replaced with STEMdiff™ Neural Induction Medium kit (StemCell, #08581) containing 10 μM Y-27632. For the subsequent 7-day induction period, the medium was changed daily to fresh, pre-warmed (37 °C) STEMdiff™ Neural Induction Medium supplemented with SMAD inhibitors (SMADi). The resulting cells exhibited typical NPC morphology and expressed definitive NPC markers of the central nervous system, including PAX6 and SOX1.

### 2.4. Immunofluorescence Staining

Cells were fixed with 4% paraformaldehyde (PFA) at room temperature for 20 min, permeabilized with 0.25% Triton X-100 for 30 min and blocked with 5% donkey serum for 1 hr before incubating with primary antibodies including OCT4 (1:200, 962649, R&D System, Minneapolis, MN, USA), NANOG (1:100, 14295-1-AP, Proteintech, Rosemont, IL, USA), PAX6 (1:200, PRB-278P, BioLegend, San Diego, CA, USA), NESTIN (1:500, 809801, BioLegend), and SOX1 (1:200, 67994-1-Ig, Proteintech) at 4 °C overnight. After extensive washing, secondary antibodies of Alexa Fluor^®^ 488 (715-545-151, Jackson ImmunoResearch, West Grove, PA, USA) and Alexa Fluor^®^ 594 (711-585-152, Jackson ImmunoResearch), together with DAPI (10 μM in 2% BSA), were added and incubated at room temperature for 4 hr. Cells were washed with PBS three times before imaging with a confocal microscope.

### 2.5. Library Construction and Sequencing for ONT

Following total RNA extraction, the integrity and quantity of the RNA were assessed using a combination of Nanodrop, Qubit, and agarose gel electrophoresis. A qualified aliquot (500 ng) was then utilized for library construction with the Oxford Nanopore cDNA PCR Barcoding Kit (SQK-PCS111, Oxford Nanopore Technologies plc, Oxford, UK). The volume was adjusted to 9 µL with Nuclease-Free Water, and first-strand cDNA synthesis was primed by oligo(dT). The resulting cDNA was subsequently amplified via PCR with LongAmp^®^ Taq DNA Polymerase (NEB). Post-amplification, the product underwent purification with AMPure beads and was then ligated to sequencing adapters harboring the motor protein. The final prepared library was loaded onto FLO-PRO002 (R9.4.1) flow cells for sequencing on a PromethION platform (Oxford Nanopore Technologies plc, Oxford, UK).

### 2.6. ONT Transcriptome Sequencing and Data Processing

Raw reads were processed using NanoFilt (v2.8.0; parameters: -q 7 -l 50) [33] to remove low-quality sequences. The full-length non-redundant transcripts were aligned to the reference genome (hg19) using Minimap2 (v2.17-r941; parameters: -ax splice -uf -k14) [34]. Novel transcripts and genes were identified by comparing the assembled transcripts against known annotations with gffcompare (v0.12.1; parameters: -R -C -K -M). Protein-coding sequences from novel transcripts were aligned to the UniProt using DIAMOND (v2.0.6.144) [35] blastp for functional annotation. Pathway annotation was performed by mapping the sequences to the Kyoto Encyclopedia of Genes and Genomes (KEGG) database using KOBAS (v3.0) [36]. Additionally, biological functions were predicted based on the relevant Gene ontology (GO) annotations of protein families recorded in the UniProt database. Gene- and transcript-level expression abundances were estimated using Salmon (v1.4.0) [37].

### 2.7. APA Analysis

A BED file containing chromosome name, strand, transcript end position, and end count was first constructed for each sample. We then used Quantifypoly(A) [38] to perform internal primer filtering (Subsequences spanning 10 nucleotides (nt) upstream and downstream of raw PASs were extracted from the corresponding genome sequences. sites with subsequences containing ≥6 consecutive adenines or ≥7 adenines within a 10 nt sliding window were defined as internal priming artifacts), PASs clustering (Initial clustering was performed with a 24 nt iterative algorithm to obtain a primary polyadenylation site cluster (PAC) pool, followed by refined sub-PAC identification using weighted density peak clustering), and annotation, finally generating a matrix containing the genomic coordinates and read counts of PASs.

Only genes containing at least two PASs with >5 reads per site were retained. The percentage of site usage (PSU) for each PAS was calculated as its read count divided by the total reads of all PASs in the same gene. To quantify changes between groups, the Differential PSU Index (DPUI) was computed as DPUI = PSU_Treat_ − PSU_Ctrl_, where PSU_Treat_ and PSU_Ctrl_ represent the average PSU of replicate samples in the treatment and control groups, respectively. Significance was assessed via the Wald test, and sites with |DPUI| ≥ 0.05 and *p* ≤ 0.05 were classified as differential APA events. For differential APA events located in 3′ UTRs, the two sites with the largest absolute DPUI values in each gene were selected to calculate the relative end offset (RED): RED = DPUI_distal_ − DPUI_proximal_. The site closer to the terminal exon was defined as the proximal PAS, and the other as the distal PAS. A RED ≥ 0.05 indicated preferential distal site usage in the treatment group, whereas RED ≤ –0.05 indicated preferential proximal site usage.

### 2.8. Illumina RNA-Seq Data Analysis

Raw reads were evaluated for quality and subsequently trimmed using FastQC (v0.11.9) [39] and Trimmomatic (v0.39) [40]. The cleaned reads were mapped to the hg19 reference genome by HISAT2 (v2.2.1) [41]. The gene-level counts were quantified using featureCounts (Unique Molecular Identifiers (UMIs) were not added during the construction of the short-read sequencing libraries). Differential expression analysis was conducted by DESeq2 (v1.38.3) [42], differential 3′ UTR-APA events were identified using DaPars (v1.0.0) [43], and differential intronic APA events were identified using IPAFinder [44].

### 2.9. Quality Control of PASs

PASs detected from ONT data were contrasted with annotated PAS in the PolyA_DB (v3.2) [45] database using BEDTools-closest (v2.30.0) [46]. The sequences within 50 nt upstream of each poly(A) site were extracted using BEDTools-getfasta, and motif enrichment analysis was performed using MEME-STREME with the following parameters: minimum width = 4, maximum width = 6, and *p*-value threshold < 0.05. Importantly, these identical parameters and thresholds were uniformly applied to assess all PAS categories.

### 2.10. Functional Enrichment Analysis

The differential APA genes were analyzed for GO and KEGG enrichment using Metascape (https://metascape.org, v3.5.20250701) with parameters set as Min Overlap = 3, *p* Value Cutoff ≤ 0.01, and Min Enrichment = 1.5. And *p*-values are calculated based on the cumulative hypergeometric distribution, and q-values are calculated using the Benjamini–Hochberg procedure to account for multiple testings. Significantly enriched terms were visualized using the ggplot2 package in R (v4.2.3).

### 2.11. Quantitative Real-Time PCR

Total RNA was extracted from cultured cells using TRIzol™ Reagent (Thermo Fisher, Waltham, MA, USA, 15596026). Reverse transcription was performed with the All-in-One 5X RT MasterMix (abm, New York, NY, USA, G592) according to the manufacturer’s protocol. Quantitative real-time PCR was carried out on an ABI Prism 7500 real-time PCR system (Applied Biosystems, Waltham, MA, USA, USA) using PowerUp™ SYBR™ Green Master Mix (Thermo Fisher, A25742). Relative expression of each gene was normalized and quantified by the 2^−ΔΔCT^ method. Primer sequences used in this study are provided in Table S2.

### 2.12. Target microRNA (miRNA) and RNA-Binding Protein (RBP) Prediction

For 3′ UTR-APA events, sequences between proximal and distal PASs were extracted using BEDTools-getfasta. Potential miRNA interactions were predicted using TargetScan (v8.0), miRDB (v6.0), and miRTarBase (v9.0), with high-confidence miRNAs defined as those identified by all three databases. RBPs and their binding motifs were predicted using the RBPDB database. For intronic APA events, sequences spanning 100 nt upstream and downstream of intronic PASs were extracted and subjected to RBP prediction using RBPDB. All results were visualized using Cytoscape (v3.10.3).

## 3. Results

### 3.1. ONT Long-Read Sequencing Reveals the Transcriptomic Diversity in Human Neural Differentiation

**Figure 1:**
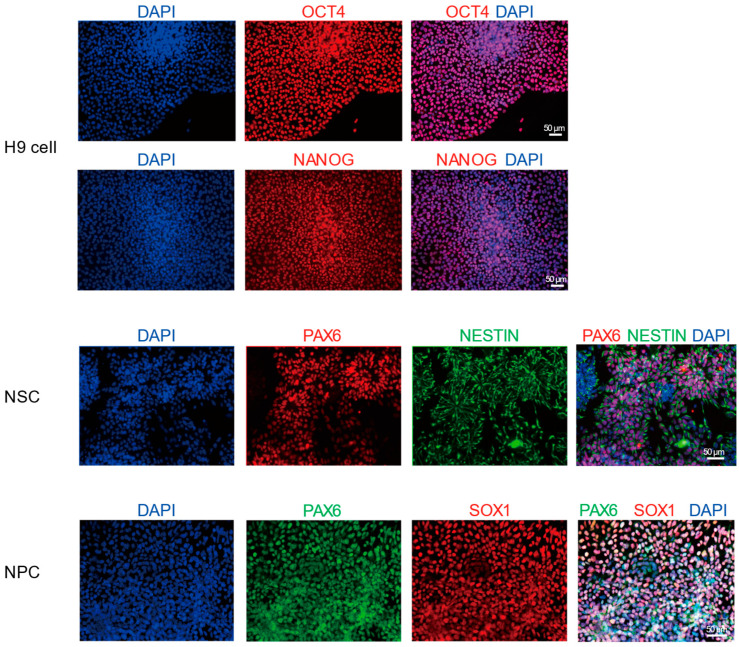
Immunofluorescence staining showing the expression of neural stem/progenitor cell markers in H9-derived cells. The cell nuclei are labeled with DAPI (blue). Nestin was detected in green. OCT4, NANOG, and SOX1 were detected in red. PAX6 was detected in red in NSCs and in green in NPCs. Scale bar = 50 μm. Original figures see File S1.

**Figure 2:**
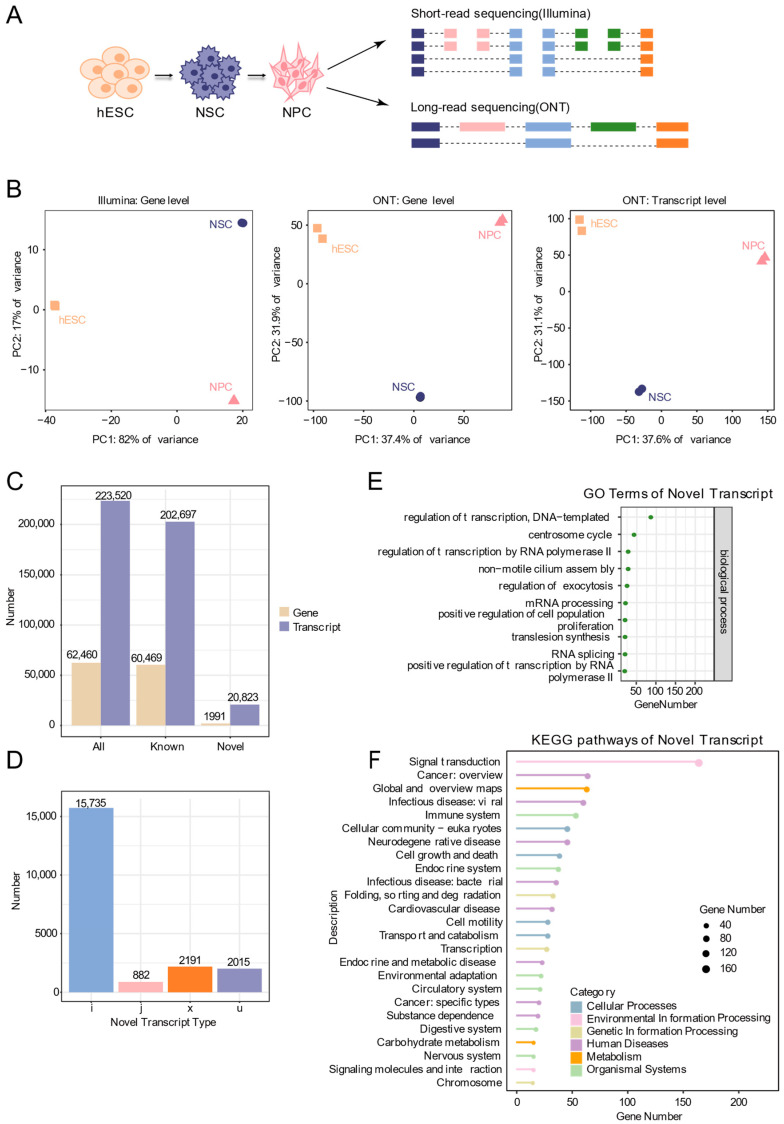
Identification of novel transcripts by ONT long-read sequencing. (**A**) Schematic plot of short-read and ONT sequencing during neural differentiation of human embryonic stem cells. (**B**) Principal component analysis based on gene expression and transcript expression. (**C**) Bar chart showing the number of known and novel genes and transcripts identified from ONT data. (**D**) Bar chart showing the types and numbers of new transcripts. Categories include: i, transcripts fully contained within introns of reference transcripts; j, transcripts with at least one matching exon; x, antisense transcripts overlapping exons of annotated genes; u, unannotated novel transcripts. (**E**) GO functional annotation of new transcripts. (**F**) KEGG pathway annotation of new transcripts.

To systematically analyze the transcriptional dynamics during the differentiation of hESCs into neural cells, this study used the hESC as the starting material. The cells were induced to differentiate into NSCs and NPCs through a directed induction protocol. During the differentiation process, immunofluorescence staining was used to detect markers specific to each stage: OCT4 and NANOG for pluripotency, PAX6 and NESTIN for NSCs, and PAX6 and SOX1 for NPCs. DAPI staining was applied to label the cell nuclei (Figure 1). The results showed that OCT4^+^ and NANOG^+^ cells accounted for approximately 98.8% and 99.0% in hESCs; PAX6^+^ and NESTIN^+^ cells for approximately 90.7% and 95.9% in NSCs; and PAX6^+^ and SOX1^+^ cells for approximately 94.3% and 95.2% in NPCs (Figure S1), confirming the efficiency and stage-specific in vitro differentiation system. Based on this, we collected cell samples at key time points and performed ONT long-read sequencing and Illumina short-read sequencing to comprehensively reveal the dynamic changes in transcripts during this differentiation process (Figure 2A, Table S1).

Two biological replicates were included for each differentiation stage. Correlation heatmaps demonstrated that intra-stage replicates clustered more closely than inter-stage samples (Figure S2), confirming the reproducibility of our experimental system. Principal component analysis (PCA) clearly separated the three stages. Notably, the separation was more distinct when using expression data derived from Nanopore sequencing compared to short-read data. Additionally, transcript-level PCA provided higher resolution than gene-level analysis, reflecting greater isoform heterogeneity during the differentiation process (Figure 2B).

Leveraging the advantages of long-read sequencing in capturing full-length transcripts, we identified 62,460 genes (60,469 known and 1991 novel), and 223,520 transcripts (202,697 known and 20,823 novel) (Figure 2C). Compared with annotated transcripts, the novel isoforms were categorized as: fully contained within introns of reference transcripts (i, 15,735), having at least one matching multi-exon (j, 882), overlapping exons on the antisense strand (x, 2191), and unknown novel transcripts (u, 2015) (Figure 2D). Functional annotations of the putative protein sequences encoded by these novel transcripts, performed with DIAMOND and KOBAS, revealed significant enrichment for processes including transcriptional regulation, mRNA processing, positive regulation of cell proliferation, and multiple pathways linked to nervous system function and neurodegenerative diseases (Figure 2E,F). These results highlight the capacity of long-read sequencing to uncover extensive transcriptomic diversity during human neural differentiation, providing a valuable resource for downstream mechanistic investigation.

### 3.2. Identification of PASs in All Gene Regions by Long-Read Sequencing

**Figure 3:**
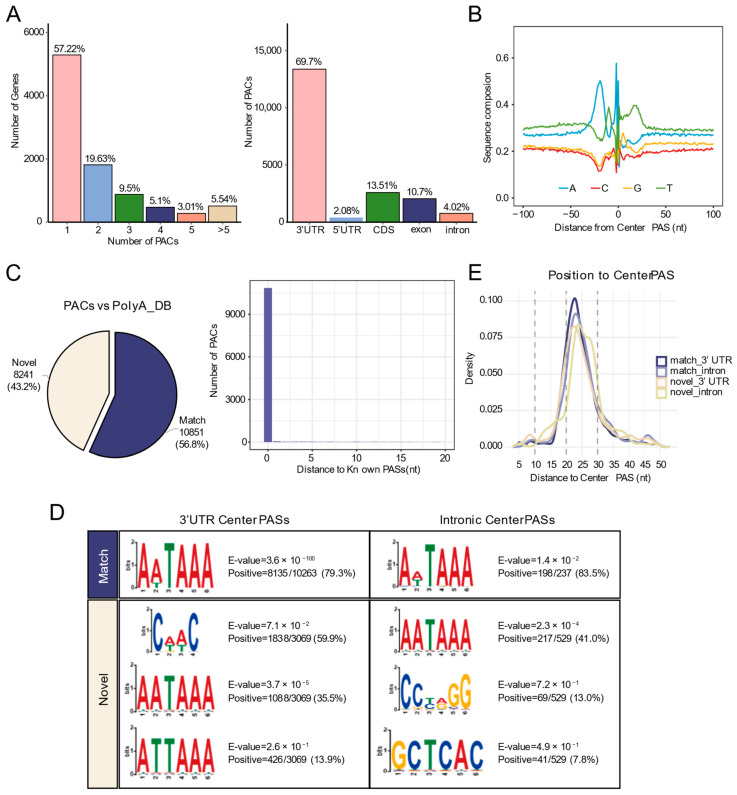
Identification of high-quality PASs by ONT long-read sequencing. (**A**) **Left**: Bar chart showing the number of genes with different numbers of PACs identified by ONT. **Right**: Bar chart showing the number of PACs in different genomic regions. (**B**) Nucleotide base distribution within 100 nt upstream and downstream of Center PASs in PACs. (**C**) **Left**: Pie chart showing the proportion of PACs identified by ONT compared to the annotated PASs from the PolyA_DB database. **Right**: Distribution of the nearest distances between PACs identified by ONT and known PASs. (**D**) Motif enrichment analysis within 50 nt upstream of center PASs. (**E**) Density distribution of the canonical PolyA signal (AATAAA) within 50 nt upstream of PASs.

We utilized QuantifyPoly(A) to identify the PACs of long-read sequencing data and designate the highest peak within each cluster as the center PAS. Across the three differentiation stages, a total of 19,175 PACs were identified. Notably, 42.78% of genes contained two or more PACs, underscoring the prevalence of APA. Genomic distribution analysis showed that PACs were predominantly located in 3′ UTRs (69.7%), followed by exon/CDS regions (24.21%), introns (4.02%), and 5′ UTRs (2.08%) (Figure 3A).

To validate the identified PACs, we examined the nucleotide composition surrounding the center PAS, which showed high similarity to reference PAS patterns (Figure 3B). Comparison with annotated sites in the PolyA_DB database revealed that 10,851 PACs perfectly matched known sites (distance = 0 nucleotides), while 8241 PACs were novel (Figure 3C). PASs are determined by the hexamer polyadenylation signal (AATAAA and its variants) and flanking regulatory elements. Motif enrichment analysis within 50 nt upstream of the center PASs showed that the canonical AATAAA signal was highly enriched in known PASs (79.3% in 3′ UTRs; 83.5% in introns). In contrast, novel 3′ UTR PASs exhibited distinct features: in addition to the AATAAA motif and its variants (AATAAA: 35.5%; ATTAAA: 13.9%), a novel motif, CAAC, was identified in 59.9%. Novel intronic PASs contained 41.0% AATAAA, as well as other motifs (13.0% CCTAGG; 7.8% GCTCAC) (Figure 3D). Density distribution analysis confirmed the expected position of AATAAA 20–30 nt upstream of the PASs (Figure 3E).

These results demonstrate that long-read sequencing data can accurately identify both known and novel PASs in all genomic regions, significantly expanding the PAS catalog.

### 3.3. Dynamic Profiles of 3′ UTR-APA During Human Neural Differentiation

**Figure 4:**
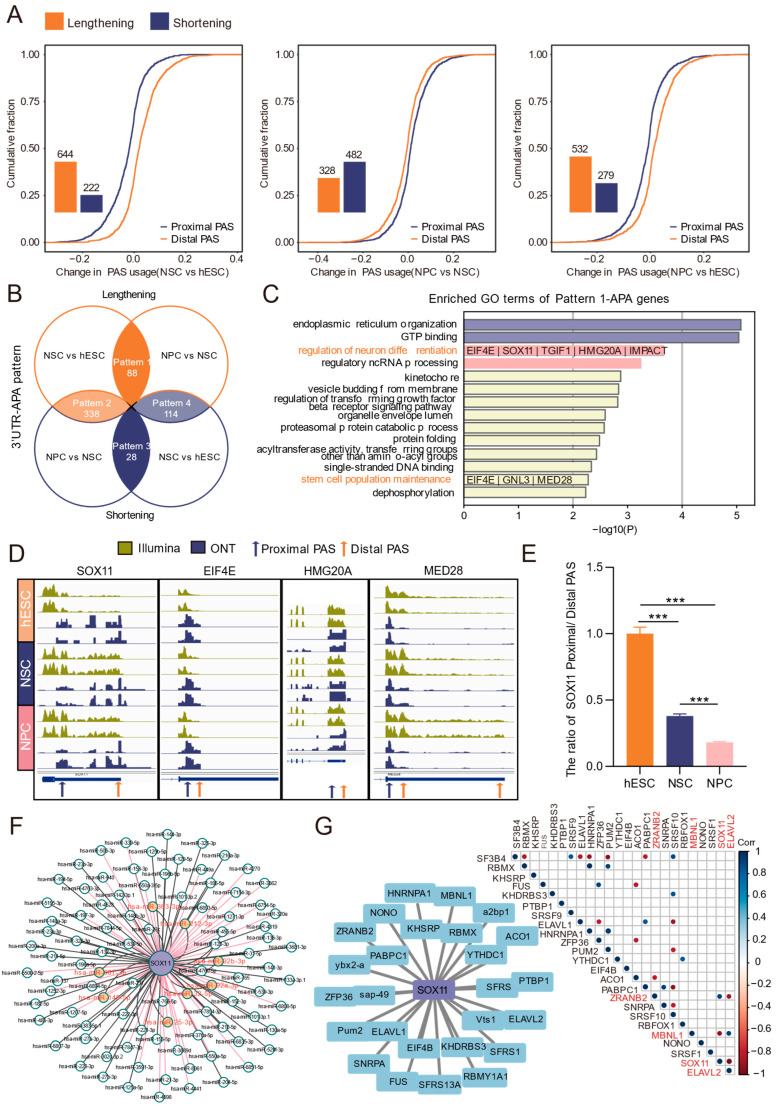
Analysis of 3′ UTR-APA dynamics during differentiation by ONT long-read sequencing. (**A**) Bar chart showing the number of differential 3′ UTR-APA events and the cumulative distribution of proximal and distal PAS usage. (**B**) Venn diagram illustrating the dynamic patterns of 3′ UTR-APA during differentiation, along with the number of genes involved. Orange represents 3′ UTR lengthening events, and blue represents shortening events in the comparison between the two groups. (**C**) Functional enrichment analysis of genes exhibiting persistent 3′ UTR lengthening (pattern 1) during differentiation. Terms associated with neural differentiation are highlighted in orange, and the corresponding enriched genes are listed below. (**D**) IGV plots of key APA-regulated genes 3′ UTR PAS usage changes validated by ONT-seq and Illumina RNA-seq data. (**E**) RT-qPCR validation of the ratio of *SOX11* proximal and distal PAS across differentiation stages. ***: *p* < 0.001. (**F**) miRNA predictions for sequences between proximal and distal PASs of *SOX11*; miRNAs predicted by TargetScan, miRDB, and miRTarBase and experimentally validated are highlighted in red. (**G**) **Left**: RBP predictions for sequences between proximal and distal PASs of *SOX11*. **Right**: Expression correlation between *SOX11* and RBPs; significant gene pairs (*p* < 0.05) are indicated by dots.

Based on long-read sequencing data, we systematically analyzed the dynamics of 3′ UTR-APA during the differentiation of hESCs into neural cells by comparing PAS usage across stages. Compared with hESCs, NSCs showed 644 3′ UTR lengthening events and 222 3′ UTR shortening events. Relative to NSCs, NPCs exhibited 328 lengthening and 482 shortening events; compared with hESCs, NPCs displayed 532 lengthening and 279 shortening events (Figure 4A).

We then intersected differential APA events from the NSCs vs. hESCs and NPCs vs. NSCs comparisons, classifying them into four dynamic patterns (Figure 4B): persistent lengthening (pattern 1), lengthening followed by shortening (pattern 2), persistent shortening (pattern 3), and shortening followed by lengthening (pattern 4). Functional enrichment analysis was performed for genes belonging to each pattern (Figure 4C, Figure S3). Pattern 1 contained 88 APA genes encompassing 175 known and 39 novel PASs (Figure 4B, Figure S4). Seven of the pattern 1 genes were enriched in the GO terms “regulation of neuron differentiation” and “stem cell population maintenance” (Figure 4C).

Visualization in IGV showed higher read abundance at distal versus proximal PASs for *SOX11*, *EIF4E*, *HMG20A*, and *MED28*, with a consistent trend in short-read data (Figure 4D). RT-qPCR showed that the proximal/distal PAS ratio of *SOX11* continuously decreased during differentiation (Figure 4E), indicating enhanced usage of distal PASs. As *SOX11* expression was progressively upregulated (Figure S5) and is known to function at multiple stages of brain development [47], we selected *SOX11* as a key APA-regulated target in neural differentiation.

Prediction of the elongated 3′ UTR region between the distal and proximal PASs of *SOX11* identified multiple potential regulatory elements. Integration of TargetScan, miRDB, and miRTarBase predictions revealed eight experimentally supported miRNAs that may target *SOX11*, such as hsa-miR-363-3p (Figure 4F). RBP binding site prediction using RBPDB suggested that multiple RBPs, such as ELAVL2, may regulate *SOX11*. Moreover, expression correlation analysis showed *SOX11* positively correlated with *ZRANB2* and negatively correlated with *MBNL1* and *ELAVL2* (Figure 4G). Together, these analyses delineate stage-specific 3′ UTR-APA patterns during neural differentiation and nominate *SOX11*, whose expression may be altered through APA-mediated post-transcriptional regulation.

### 3.4. Widespread Intronic APA Events in the Neural Progenitor Cell Stage

**Figure 5:**
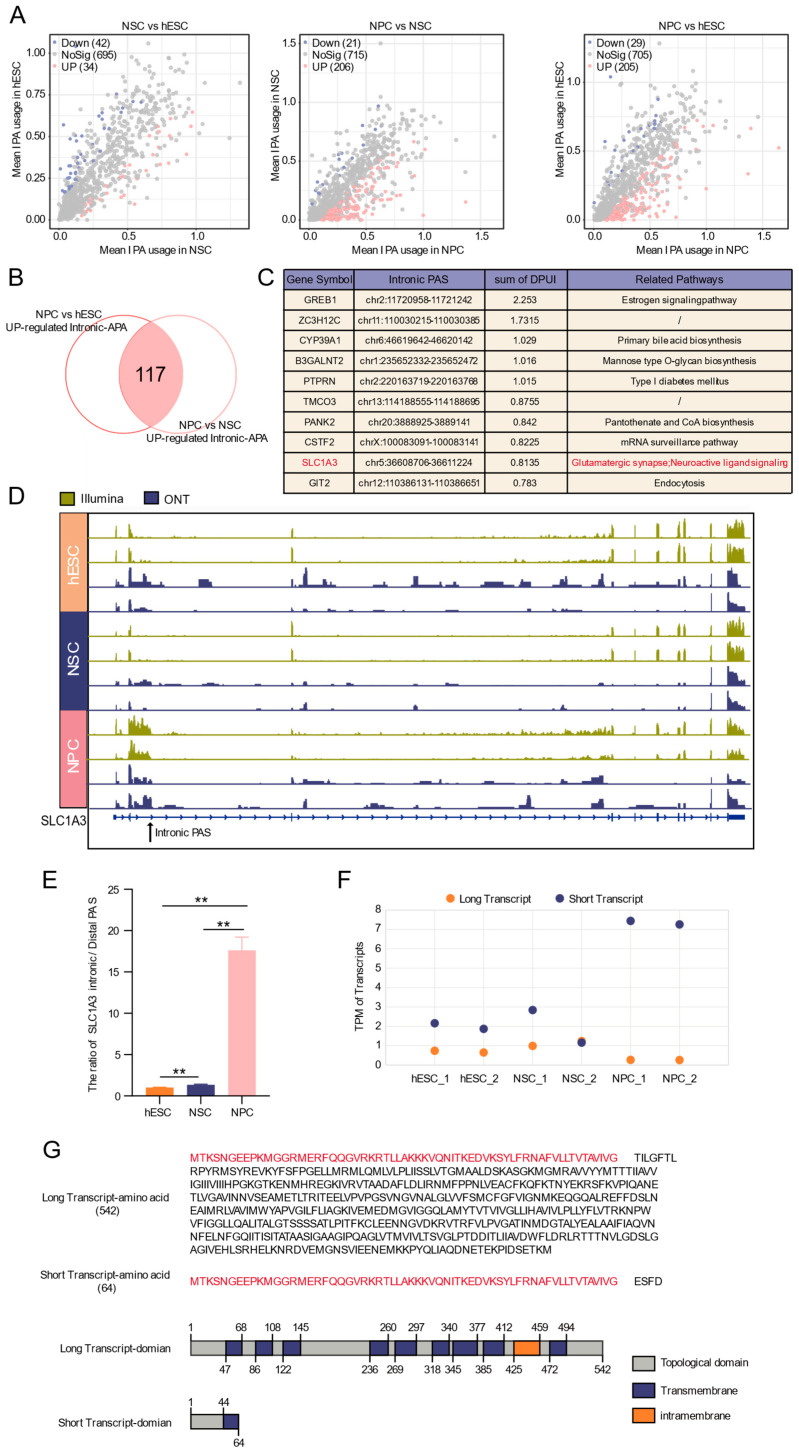
Dynamics of intronic APA during neural differentiation. (**A**) Volcano plot of differential intronic APA events. (**B**) Venn diagram of intronic APA events specifically up-regulated at the neural progenitor stage. (**C**) Top ten up-regulated intronic APA events and their functional annotations. (**D**) IGV plot of *SLC1A3* intronic PAS usage changes validated by ONT-seq and Illumina RNA-seq data. (**E**) RT-qPCR validation of the ratio of *SLC1A3* intronic and most distal PAS across developmental stages. **: *p* < 0.01. (**F**) The expression profiles of long and short *SLC1A3* transcript isoforms across developmental stages, as quantified by ONT sequencing data. (**G**) Prediction of protein domains for full-length and truncated *SLC1A3* transcripts isoforms.

Given that transcripts generated by intronic APA are typically low in abundance and that fewer intronic PASs were identified by ONT long-read sequencing, we used high-throughput short-read sequencing data and the corresponding tool IPAFinder to identify differentially intronic APA events, with validation using long-read data. Comparative analysis revealed that, compared with hESCs, 34 upregulated and 42 downregulated intronic PASs were identified in NSCs. Compared with NSCs, 206 intronic PASs were upregulated and only 21 were downregulated in NPCs. Similarly, compared with hESCs, 205 intronic PASs were upregulated and only 29 were downregulated in NPCs (Figure 5A). The number of intronic PASs upregulated at the NPC stage is approximately six times that at the NSC stage, indicating a stage-specific burst of intronic APA.

Focusing on this NPC-specific enrichment, we intersected upregulated intronic PASs from the NPC vs. hESC and NPC vs. NSC comparisons, identifying 117 NPC-specific intronic APA events (Figure 5B). Ranking by the degree of change (DPUI) highlighted the top ten genes, including *SLC1A3*, which is associated with neural pathways (Figure 5C) and nervous system development [48].

IGV visualization using both sequencing data confirmed the specific usage of an intronic PAS in *SLC1A3* in NPCs, characterized by increased read abundance at the intronic site and upstream region, coupled with a decrease in the downstream region (Figure 5D), suggesting the potential generation of truncated transcripts in *SLC1A3*. RT-qPCR verified that the intronic/distal PAS usage ratio for *SLC1A3* was significantly higher in NPCs than in hESCs or NSCs (Figure 5E). Quantification further showed that the short transcript increased while the full-length transcript decreased in NPCs (Figure 5F), confirming enhanced intronic PAS usage. Structural prediction indicated that the truncated *SLC1A3* transcript lacks several key domains present in the full-length isoform (Figure 5G), raising the possibility that intronic APA generates transcript isoforms with predicted structural alterations. Prediction of RBP binding sites within 100 nt flanking this intronic PAS suggested potential regulation by factors such as SNRPA and PABPC1 (Figure S6).

Together, these results demonstrate a pronounced, stage-specific activation of intronic APA during the NPC stage and identify *SLC1A3* as a representative gene exhibiting APA-mediated isoform switching associated with neural differentiation.

### 3.5. Accurate Identification of Exonic PASs by Long-Read Sequencing

**Figure 6:**
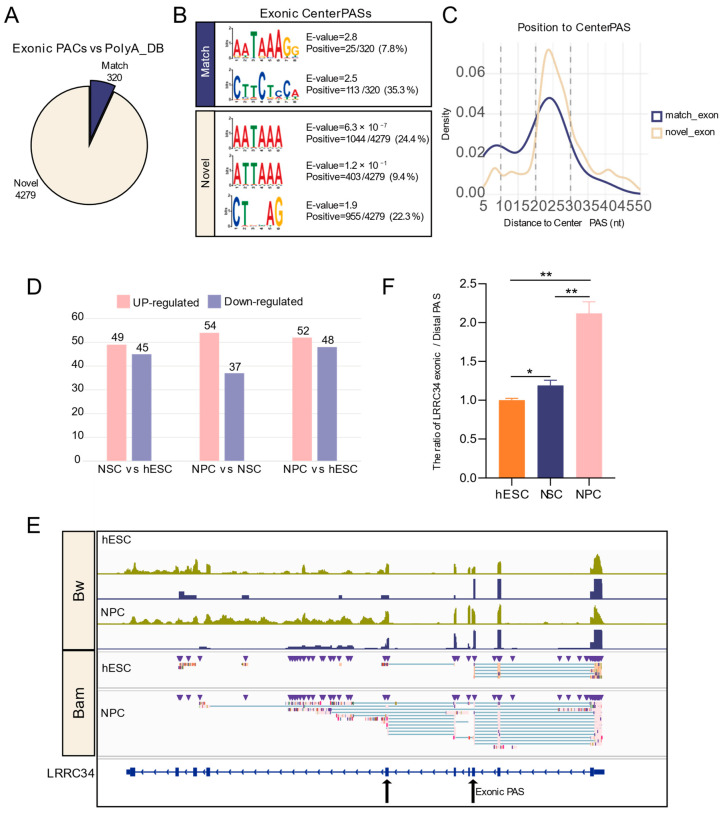
Identification and dynamics analysis of exonic PASs during neural differentiation. (**A**) Pie chart showing the proportion of exonic PACs identified by ONT compared to the annotated PASs in the PolyA_DB database. (**B**) Motif enrichment within 50 nt upstream of center PASs in PACs. (**C**) Density distribution of the canonical PolyA signal (AATAAA) within 50 nt upstream of exonic PASs. (**D**) Bar chart showing the number of significant differential exonic APA events. (**E**) IGV plot validating changes in exonic PAS usage by ONT-seq and Illumina RNA-seq data; upper panels show data in bw format, and lower panels show data in ONT-bam format. (**F**) RT-qPCR validated the ratio of *LRRC34* exonic and most distal PAS across developmental stages. *: *p* < 0.05; **: *p* < 0.01.

Exon APA can alter the mRNA coding sequence and 3′ UTR, directly impacting the resulting protein sequence and, potentially, its function. Using long-read sequencing, we identified 4599 exonic PACs. Comparison with the PolyA_DB database showed that the vast majority (4279 PACs, 93.0%) were novel and unannotated sites (Figure 6A), underscoring the limited annotation of exonic PASs in existing resources.

We then examined the sequence features supporting these PASs. Motif enrichment analysis within 50 nt upstream of the center PASs showed that among the 320 matched PASs, the canonical AATAAA signal was infrequent (7.8%), while a novel motif CTTCTCCA was highly enriched (35.3%). Interestingly, in the novel PASs, the canonical AATAAA signal was more frequent (24.4%), and its variant ATTAAA was also prevalent (9.4%) (Figure 6B). Density distribution analysis confirmed that AATAAA was positioned 20–30 nt upstream of the PASs (Figure 6C), supporting their authenticity.

We next examined the dynamic usage of exonic PASs during differentiation. Compared with hESCs, NSCs showed 49 upregulated and 45 downregulated exonic PASs; compared with NSCs, NPCs had 54 upregulated and 37 downregulated PASs; and compared with hESCs, NPCs exhibited 52 upregulated and 48 downregulated PASs (Figure 6D). Visualization of representative genes in IGV revealed a gradual decrease in read coverage within exons, accompanied by an increase in truncated transcripts terminating at exonic PASs (Figure 6E). For *LRRC34*, RT-qPCR confirmed a significantly increased exonic PAS/distal PAS usage ratio at the NPC stage (Figure 6F), indicating a preferential switch from the distal 3′ UTR PAS to the exonic PAS.

These results suggest that long-read sequencing could serve as a robust tool for precisely identifying exonic PASs. Our analysis not only expanded the catalog of exonic PAS but also captured their dynamic regulation, advancing the understanding of APA-driven transcriptome diversity.

## 4. Discussion

While high-throughput short-read sequencing is widely used in APA studies, its limited read length complicates accurate reconstruction of full-length RNA transcripts, especially given the complexity of the human transcriptome. In contrast, single-molecule long-read sequencing directly captures complete transcripts including poly(A) tails, enabling precise identification of PASs and quantification of their usage without assembly. However, the relatively low throughput of current long-read sequencing results in lower coverage than short-read sequencing [30,49,50], which restricts the detection and quantification of low-abundance isoforms and PASs, particularly in complex genomic regions such as introns. Therefore, in this study, we combined the complementary strengths of ONT long-read sequencing and Illumina short-read sequencing to systematically compare the differential usage of PASs in different gene regions across three stages: hESC, NSC, and NPC, and construct a comprehensive dynamic map of APA.

Through ONT sequencing, we re-annotated the transcriptomes of the three stages of neural differentiation, identifying a total of 223,520 transcripts, including 20,823 novel transcripts. These newly identified transcripts are predicted to encode functional proteins linked to transcriptional regulation, mRNA processing, and nervous system diseases. Prior large-scale efforts, such as the GTEx project, reported 71,735 novel transcripts across diverse tissues [51], and a long-read study of colorectal cancer identified 56,790 novel isoforms [52]. Although our sample size is more limited, long-read sequencing here revealed substantially more novel transcripts than typically detected by short-read approaches in comparable studies [53], significantly expanding the known transcript diversity during neural differentiation and enabling deeper investigation of post-transcriptional regulatory mechanisms.

PAS identification relies on characteristic sequence motifs in flanking regions. Upstream conserved elements primarily include the canonical AAUAAA hexanucleotide sequence and its variants, UGUA motifs, and oligo(U) stretches. Downstream regulatory elements typically consist of GU-rich and oligo(U) sequences. Among these, AAUAAA serves as the core signal, widely distributed upstream of most PAS, and helps distinguish strong from weak PASs [54]. Using ONT sequencing data, we identified 19,175 PACs distributed across 3′ UTRs, exons, introns, and 5′ UTRs, including 8241 novel PACs. The sequences 50 nt upstream of these novel PASs significantly enriched approximately 40% of the canonical AATAAA signal and its variants, located within 20–30 nt upstream of the PASs, consistent with previous studies. These novel PASs may introduce new cis-regulatory elements such as miRNAs and RBPs, uncovering APA events missed in earlier studies. Thus, ONT sequencing provides an accurate and expanded map of PASs during neural differentiation.

We next systematically analyzed the dynamic usage of PASs across genomic regions during differentiation. To evaluate the impact of sequencing technologies on APA profiling, we first compared PAS abundance between the long-read and short-read platforms. As expected, systematic differences in absolute PAS counts were observed (Figure S7A), attributable to their distinct technical principles and quantification biases. However, when focusing on differential APA events detected by both platforms, the DPUI values from the same stage showed significant positive cross-platform correlation across different comparisons (Figure S7B), indicating consistent capture of APA dynamics. Events identified by only one sequencing platform can reflect the distinct characteristics of each technology. For example, short-read sequencing data revealed an intronic APA event in the gene *ZC3H12C* at the NPC stage, whereas this event was missed in ONT data due to negligible read coverage of this gene (Figure S7C). Such cases illustrate the technical complementarity between platforms. Incorporating these platform-specific findings can help construct a more comprehensive map of APA regulation.

Based on the switch between proximal and distal PAS usage during differentiation, we classified 3′ UTR-APA events into four dynamic patterns. Pattern 1 (persistent lengthening) is associated with neuronal differentiation regulation and stem-cell maintenance, potentially playing a key role in cell fate determination. Pattern 2 (lengthening followed by shortening) involves genes related to mitochondrial organization and cell-cycle regulation. Pattern 3 (persistent shortening) is enriched for genes functioning in protein homeostasis. Pattern 4 (shortening followed by lengthening) includes genes involved in Golgi transport, lysosomal organization, and translation. These stage-specific APA patterns likely allow precise, temporally controlled regulation of different functional modules, ensuring orderly differentiation. Focusing on Pattern 1, we identified *SOX11* and predicted miRNAs and RBPs that may bind to its alternative 3′ UTR. *SOX11*, a member of the *SoxC* transcription factor family and plays an important regulatory role in early embryonic development. Knockout of *Sox11* leads to perinatal death in mice, accompanied by severe developmental defects [55,56]. Notably, *SOX11* drives neurogenesis by promoting the differentiation of embryonic stem cells into neural precursors and the generation of mature neurons and glial cells [57,58]. Its expression is known to be modulated by post-transcriptional mechanisms, including miRNA-mediated regulation [59,60]. In line with this, Eldon E Geisert et al. reported sustained up-regulation of *Sox11* after regenerative therapy in damaged retinas, with a longer 3′ UTR isoform being specifically elevated in late-stage glaucoma [61]. This supports the notion that distal PAS selection in *SOX11* may introduce additional miRNA/RBP-binding sites, thereby enhancing mRNA stability and contributing to its up-regulation during neural differentiation.

While previous studies of APA in neural differentiation have largely focused on 3′ UTRs, our work extends the dynamic map to upstream regions (UR-APA), revealing intronic and exon APA events during the differentiation of hESCs into NPCs. Since long-read sequencing coverage is biased toward abundant transcripts, we leveraged short-read data and dedicated tools to analyze intronic PAS usage. We found a substantial number of intronic PASs usage upregulation events specifically enriched in the NPC stage. This is consistent with previous studies that UR-APA is typically upregulated in proliferating cells and suppressed upon differentiation. Previous studies have also shown that the RNA-associated protein Srrt/Ars2 maintains the characteristics of embryonic stem cells by inhibiting premature termination at intronic PASs in a large number of transcripts [62]. La Rosa et al. found that the RBP Sam68 prevents the recognition and premature termination of *Aldh1a3* pre-mRNA by binding to its intronic PAS, thereby facilitating the expression of the metabolic enzyme ALDH1A3 and regulating the fate of neural precursor cells [63]. Among the genes with upregulated intronic PASs in NPCs, we identified *SLC1A3*, which is a member of the solute carrier family 1 (*SLC1*) and encodes the excitatory amino acid transporter 1 (EAAT1). EAAT1 is responsible for the transport of aspartate and glutamate across the cell membrane, maintaining microenvironmental homeostasis. Studies have shown that *SLC1A3* is highly expressed in adult neural progenitor and stem cells, affecting cell self-renewal and multilineage differentiation capabilities induced by glutamate [64,65,66]. In NPCs, the intronic PAS of *SLC1A3* was upregulated, accompanied by decreased read abundance downstream, suggesting active production of a truncated transcript. Protein domain prediction indicated that the truncated protein isoforms lacked several key functional domains present in the full-length protein. We therefore propose that in NPCs, *SLC1A3* may undergo intronic APA, possibly regulated by RBPs, leading to truncated isoforms that could reduce functional EAAT1 levels. This suggests a new possibility of influencing the glutamate signaling microenvironment through post-transcriptional regulation. However, the exact protein function remains to be confirmed by subsequent experiments.

Additionally, through ONT sequencing, we identified 4599 exonic PASs, of which 4279 were novel when compared to PolyA_DB. Within 50 nt upstream of these novel PASs, we successfully enriched the canonical PolyA signal AATAAA and its variant ATTAAA. The lower proportion of AATAAA motifs in exonic PAS (match PASs: 7.8%, novel PASs: 24.4%) compared to those in 3′ UTR and intron regions may explain the rarity of exon APA events. Subsequent analysis of exonic PAS usage across different stages revealed a number of differentially regulated exon APA genes. For example, in *LRRC34*, IGV visualization and RT-qPCR confirmed preferential usage of an internal exonic PAS at the NPC stage. This usage leads to skipping of exon 6 and direct termination at exon 3, deleting the downstream coding sequence.

By integrating long- and short-read sequencing, this study provides a detailed, dynamic map of PAS usage during embryonic stem cell neural differentiation. The map offers insight into how APA participates in stem cell fate and neural differentiation and serves as a valuable resource for subsequent functional studies. In the future, it is necessary to continue developing APA analysis tools specifically for ONT long-read sequencing data to more effectively uncover all types of APA events and conduct functional validation of key APA events to elucidate the post-transcriptional regulatory networks during differentiation.

## 5. Conclusions

In summary, our study identified 20,823 novel transcripts and 8241 PASs through long-read sequencing, significantly expanding the understanding of the complexity of the transcriptome during neural differentiation. Subsequently, by integrating the complementary strengths of long- and short-read sequencing, we systematically elucidated the stage-specific regulatory patterns of APA in 3′ UTR, intron, and exon regions. Notably, we revealed four dynamic patterns of 3′ UTR-APA during differentiation and specific enrichment of intronic APA events in the NPC stage, which was approximately six times higher than that in the NSC stage. We also identified key genes such as *SOX11* and *SLC1A3*, whose APA events may play crucial roles in neural differentiation. These findings provide direct molecular clues and a mechanistic framework for future studies on the mechanisms of neural development and related diseases at the post-transcriptional level.

## Data Availability

The raw sequence data reported in this paper have been deposited in the Genome Sequence Archive (https://ngdc.cncb.ac.cn/gsa-human, accessed on 19 December 2025, GSA-Human: HRA013834) and are available upon reasonable request to the corresponding authors.

## Supplementary Materials

The following supporting information can be downloaded at: https://www.mdpi.com/article/10.3390/biology15010024/s1.
