# Supplementary material for: Stage-Specific Alternative Polyadenylation During Human Neural Differentiation Revealed by Integrated Long- and Short-Read Sequencing
Source: Biology (Basel). 2025 Dec 23;15(1):24. doi: 10.3390/biology15010024 (PMC12784838; doi:10.3390/biology15010024)
Supplement: Supplementary file 1 [file biology-15-00024-s001.zip › FigureS3.pdf]

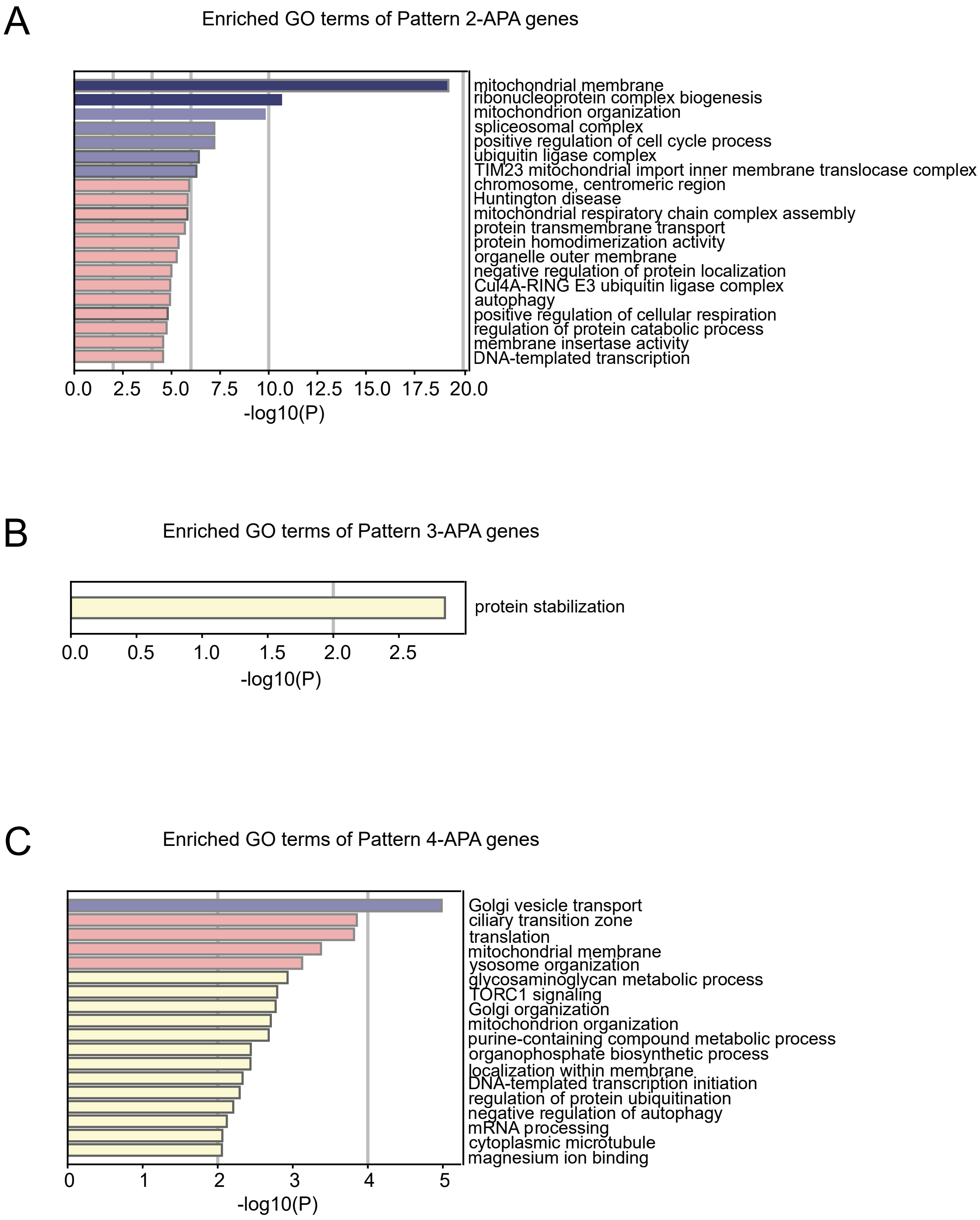
A
Enriched GO terms of Pattern 2-APA genes
mitochondrial membrane
ribonucleoprotein complex biogenesis
mitochondrion organization
spliceosomal complex
positive regulation of cell cycle process
ubiquitin ligase complex
TIM23 mitochondrial import inner membrane translocase complex
chromosome, centromeric region
Huntington disease
mitochondrial respiratory chain complex assembly
protein transmembrane transport
protein homodimerization activity
organelle outer membrane
negative regulation of protein localization
Cul4A-RING E3 ubiquitin ligase complex
autophagy
positive regulation of cellular respiration
regulation of protein catabolic process
membrane insertase activity
DNA-templated transcription
0.0 2.5 5.0 7.5 10.0 12.5 15.0 17.5 20.0
-log10(P)
B
Enriched GO terms of Pattern 3-APA genes
protein stabilization
0.0 0.5 1.0 1.5 2.0 2.5
-log10(P)
C
Enriched GO terms of Pattern 4-APA genes
Golgi vesicle transport
ciliary transition zone
translation
mitochondrial membrane
ysosome organization
glycosaminoglycan metabolic process
TORC1 signaling
Golgi organization
mitochondrion organization
purine-containing compound metabolic process
organophosphate biosynthetic process
localization within membrane
DNA-templated transcription initiation
regulation of protein ubiquitination
negative regulation of autophagy
mRNA processing
cytoplasmic microtubule
magnesium ion binding
0 1 2 3 4 5
-log10(P)
