# Supplementary figures and images for: Stage-Specific Alternative Polyadenylation During Human Neural Differentiation Revealed by Integrated Long- and Short-Read Sequencing
Source: Biology (Basel). 2025 Dec 23;15(1):24. doi: 10.3390/biology15010024 (PMC12784838; doi:10.3390/biology15010024)

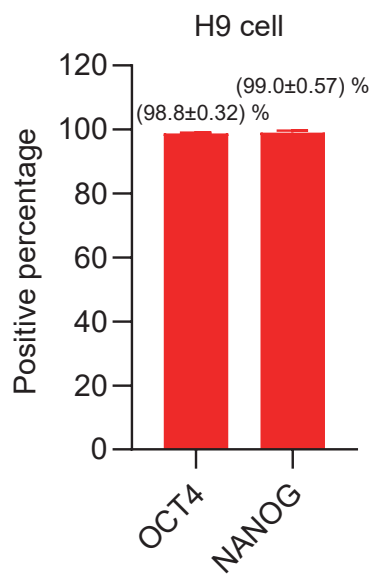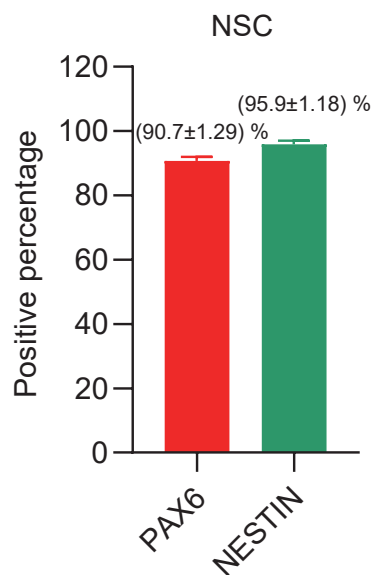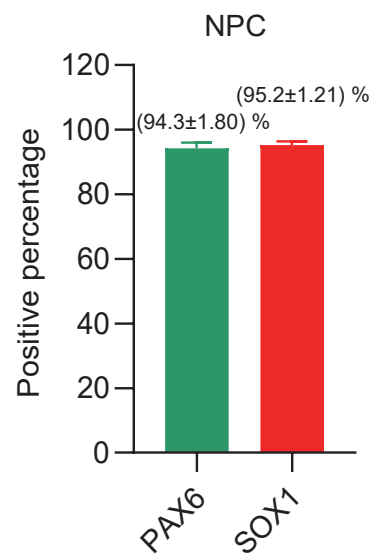

Supplement: Supplementary file 1 [file biology-15-00024-s001.zip › FigureS1.pdf]

Illumina: Gene level

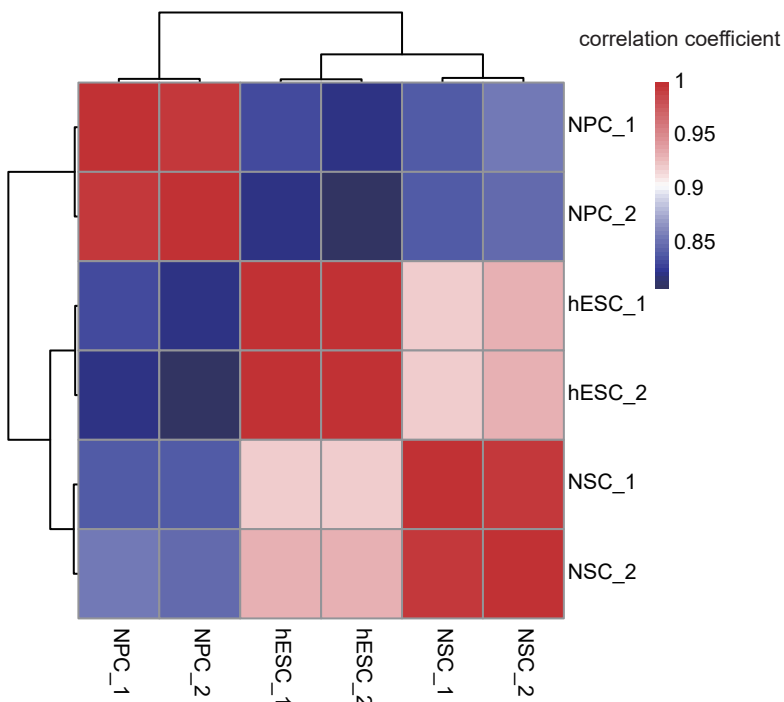

ONT: Gene level

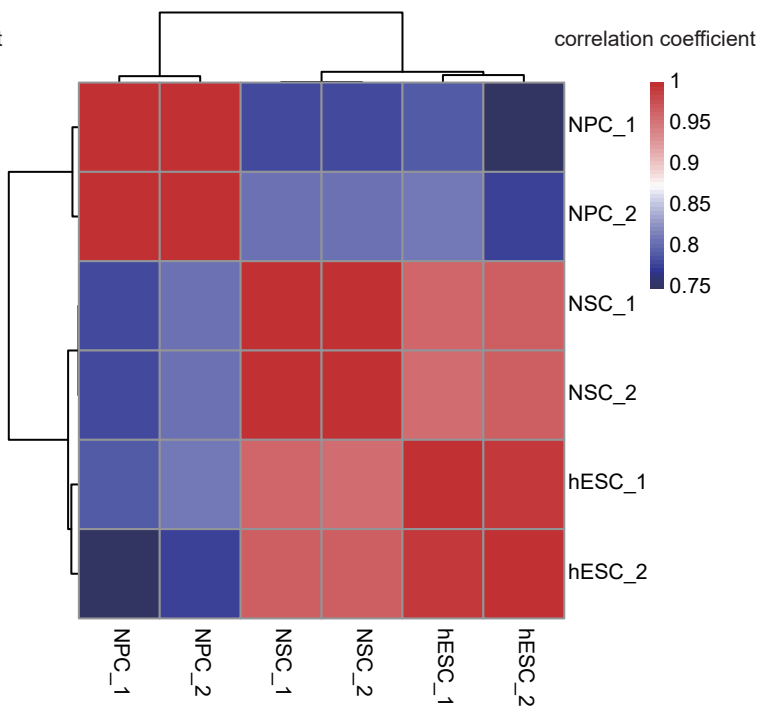

Supplement: Supplementary file 1 [file biology-15-00024-s001.zip › FigureS2.pdf]

A

3'UTR PACs vs PolyA\_DB

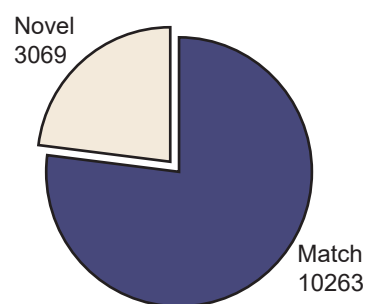

B

Lengthening 3'UTR PACs vs PolyA\_DB

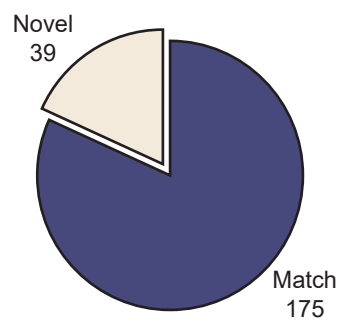

Supplement: Supplementary file 1 [file biology-15-00024-s001.zip › FigureS4.pdf]

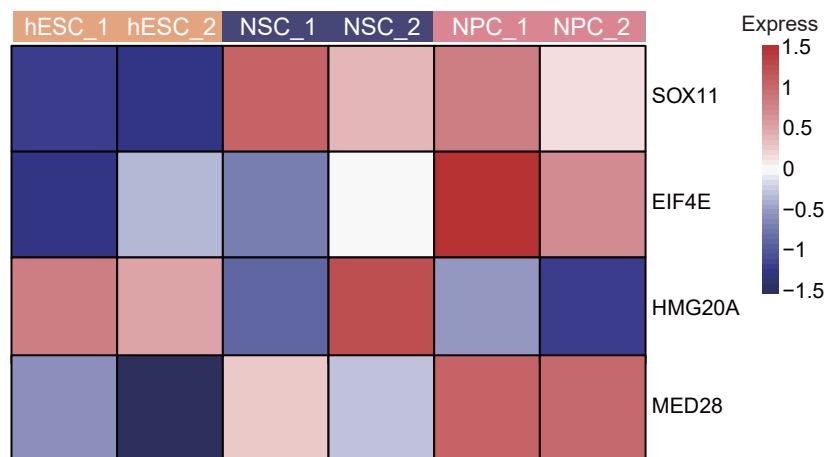

Supplement: Supplementary file 1 [file biology-15-00024-s001.zip › FigureS5.pdf]

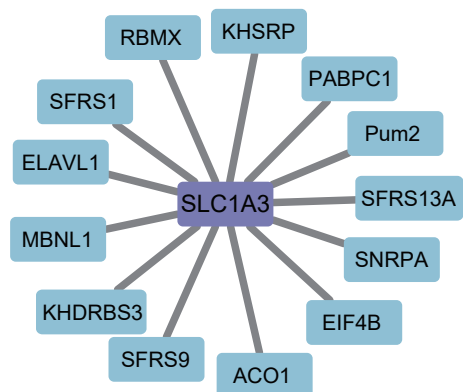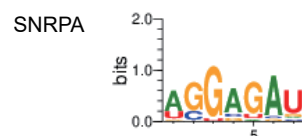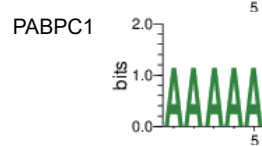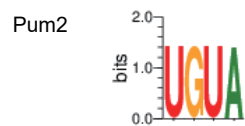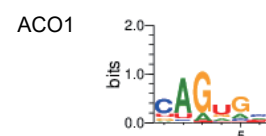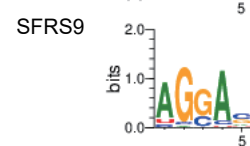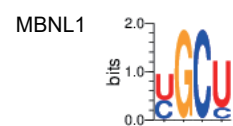

Supplement: Supplementary file 1 [file biology-15-00024-s001.zip › FigureS6.pdf]

A

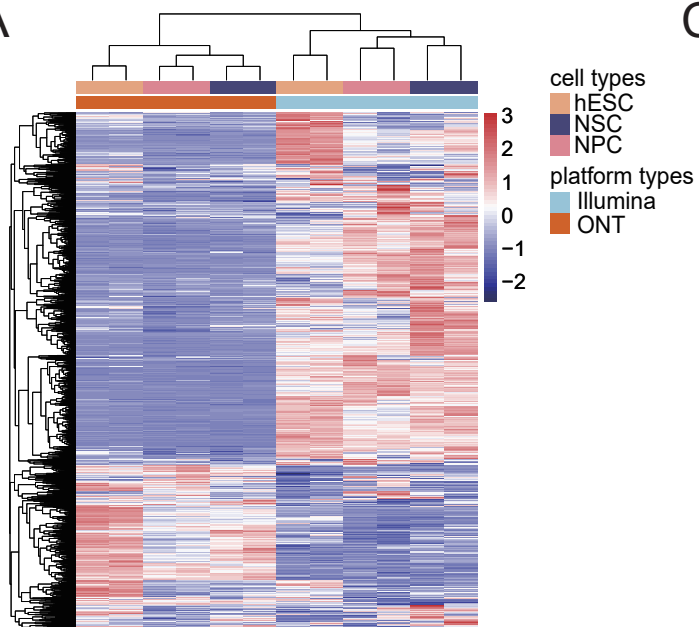

C

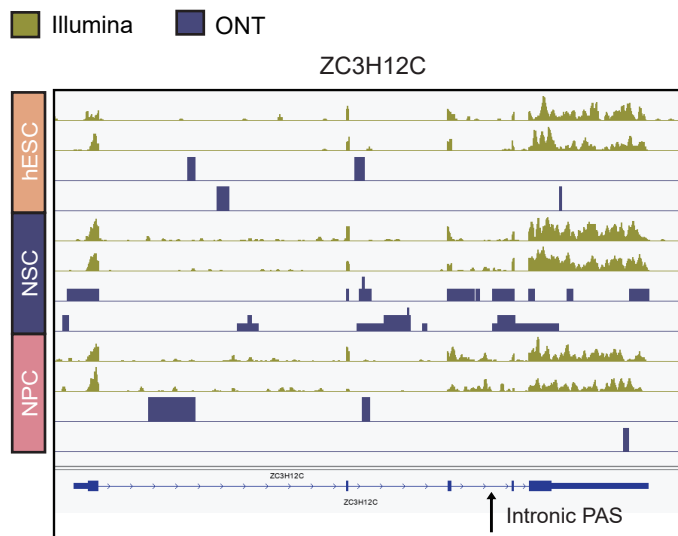

B

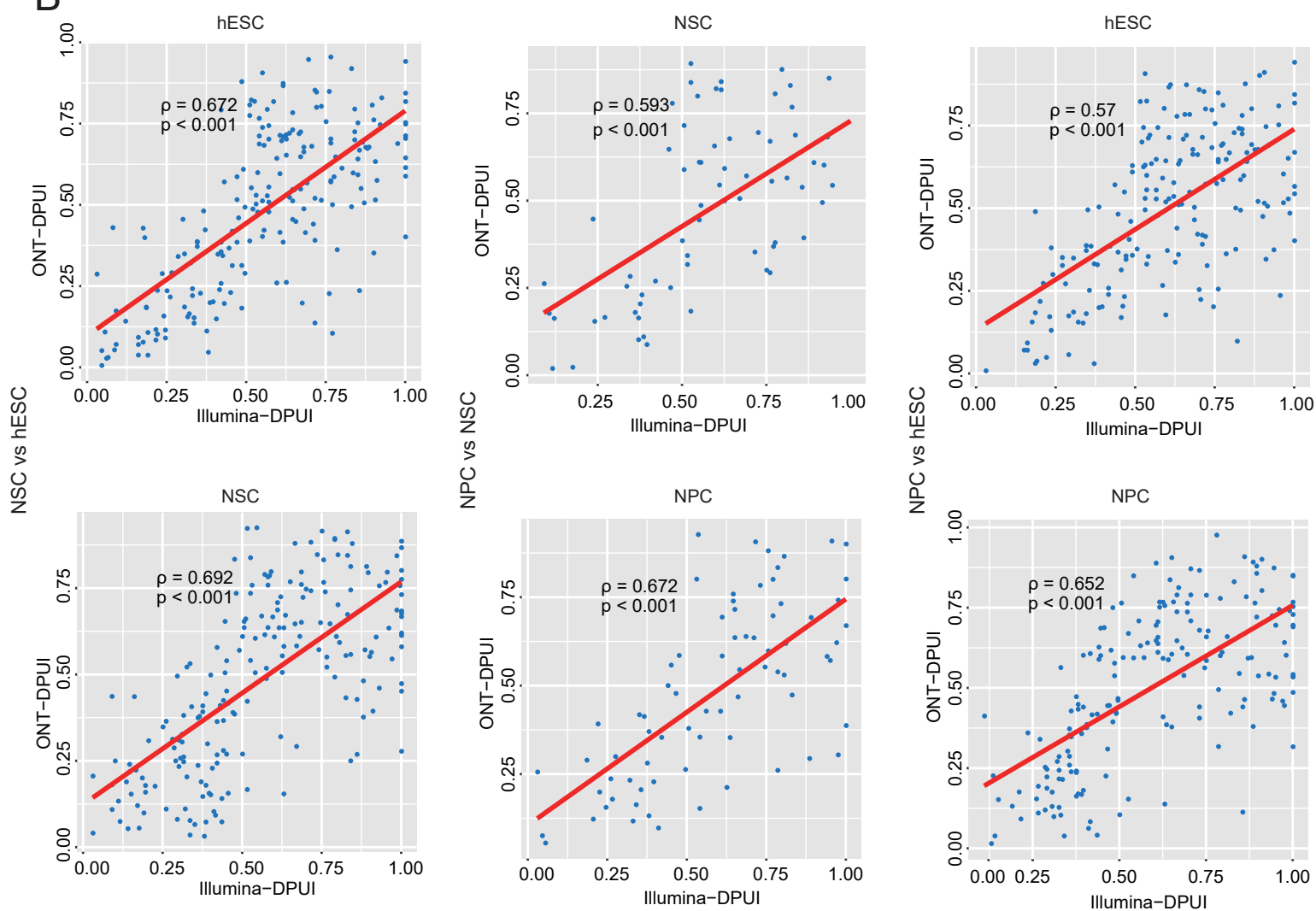

Supplement: Supplementary file 1 [file biology-15-00024-s001.zip › FigureS7.pdf]

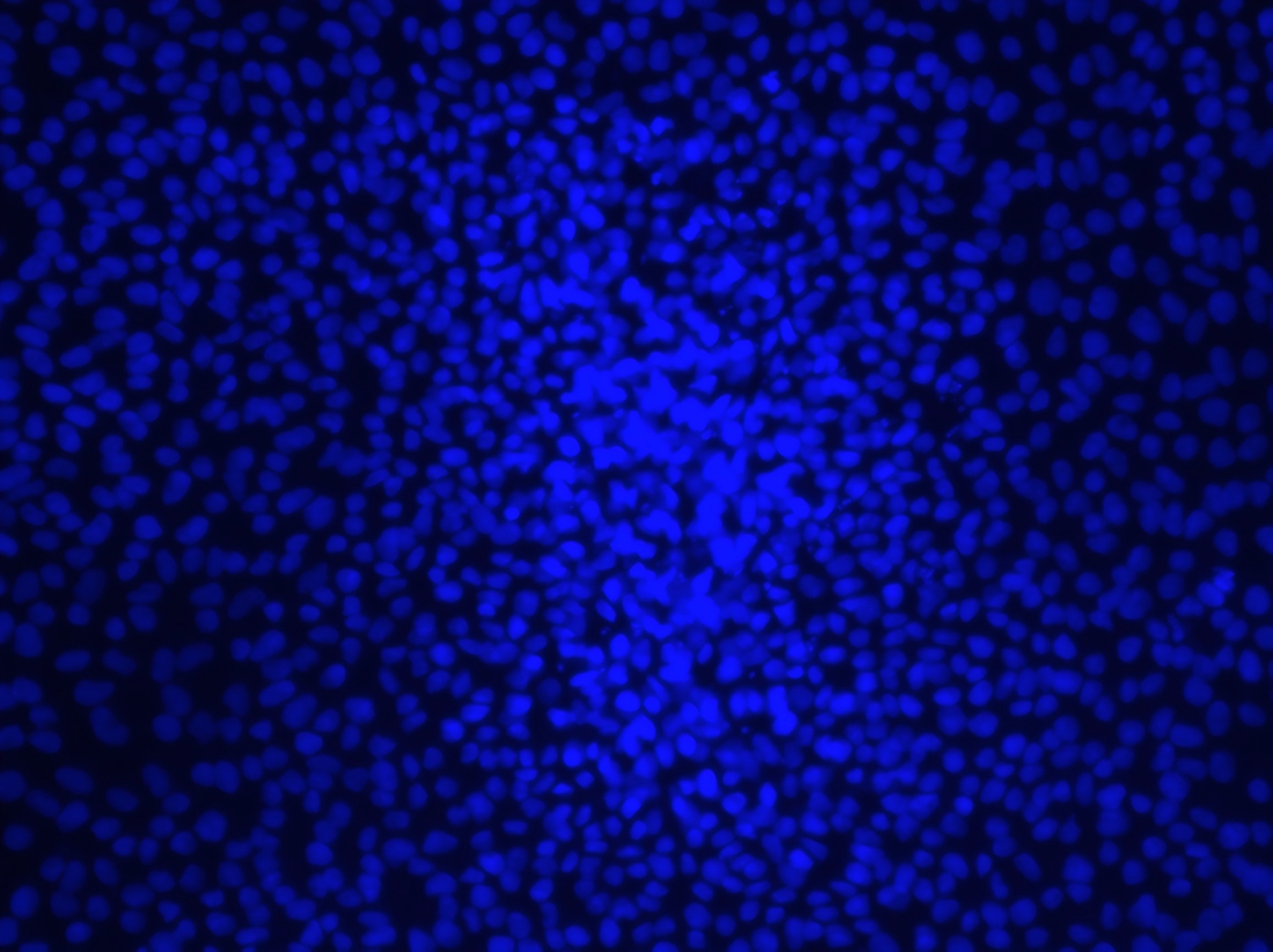

Supplement: Supplementary file 1 [file biology-15-00024-s001.zip › File S1. Figure1-original images/H9-NANOG/DAPI.png]

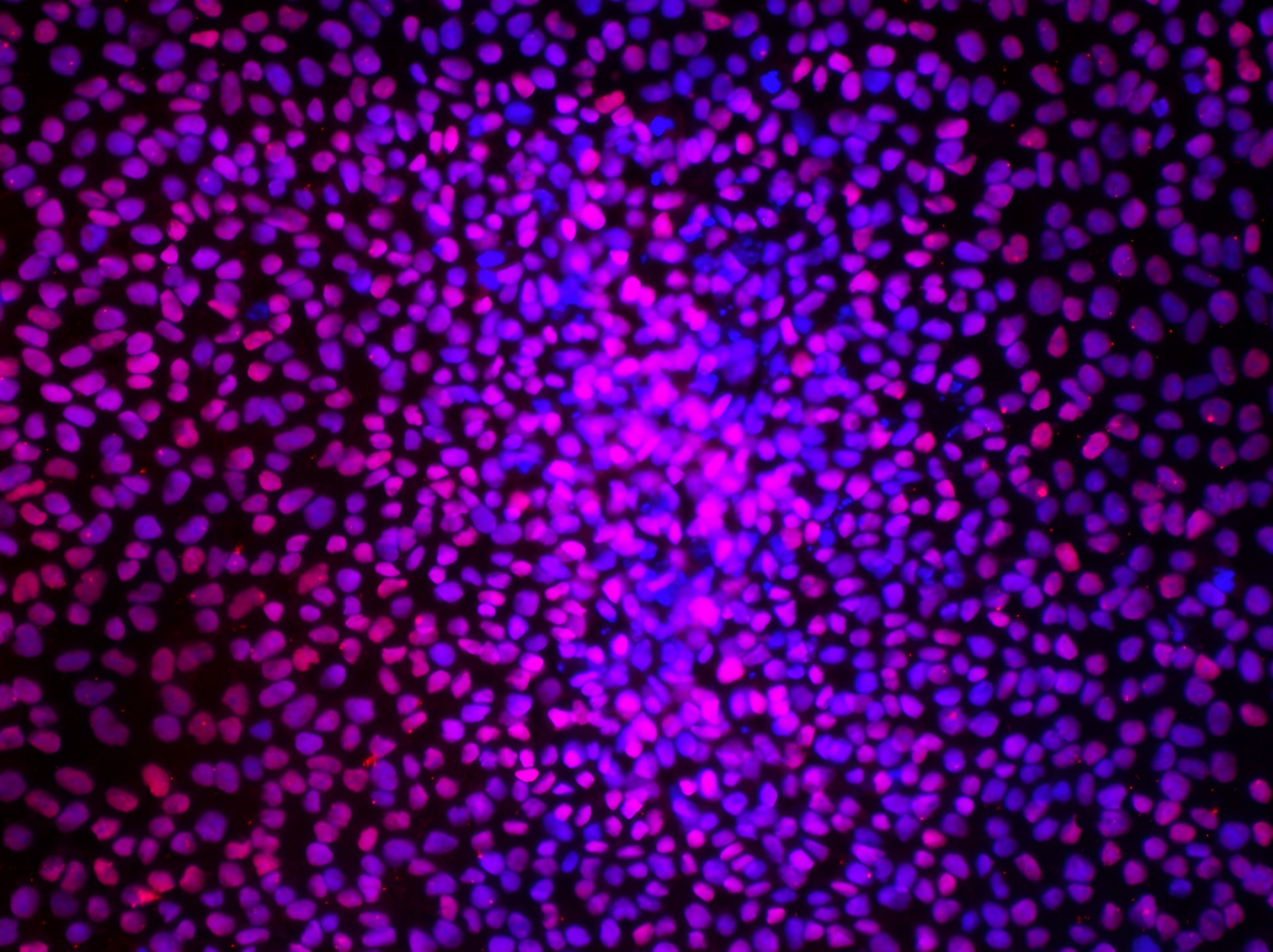

Supplement: Supplementary file 1 [file biology-15-00024-s001.zip › File S1. Figure1-original images/H9-NANOG/NANOG-DAPI.png]

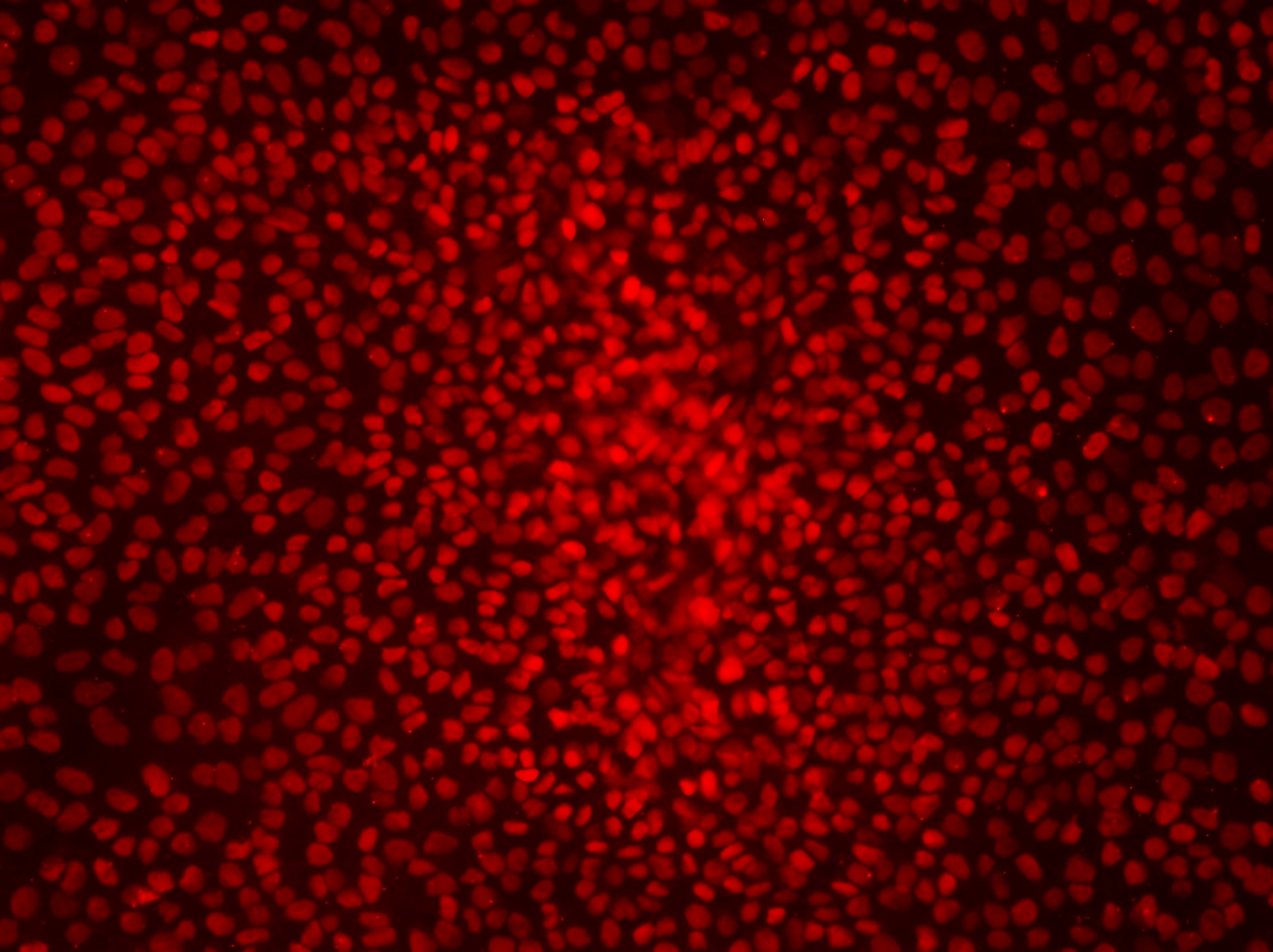

Supplement: Supplementary file 1 [file biology-15-00024-s001.zip › File S1. Figure1-original images/H9-NANOG/NANOG.png]

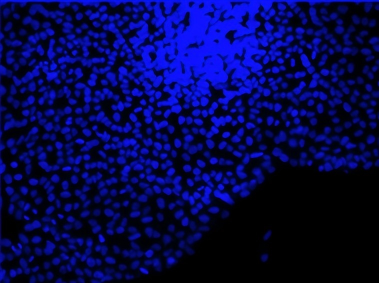

Supplement: Supplementary file 1 [file biology-15-00024-s001.zip › File S1. Figure1-original images/H9-OCT4/DAPI.png]

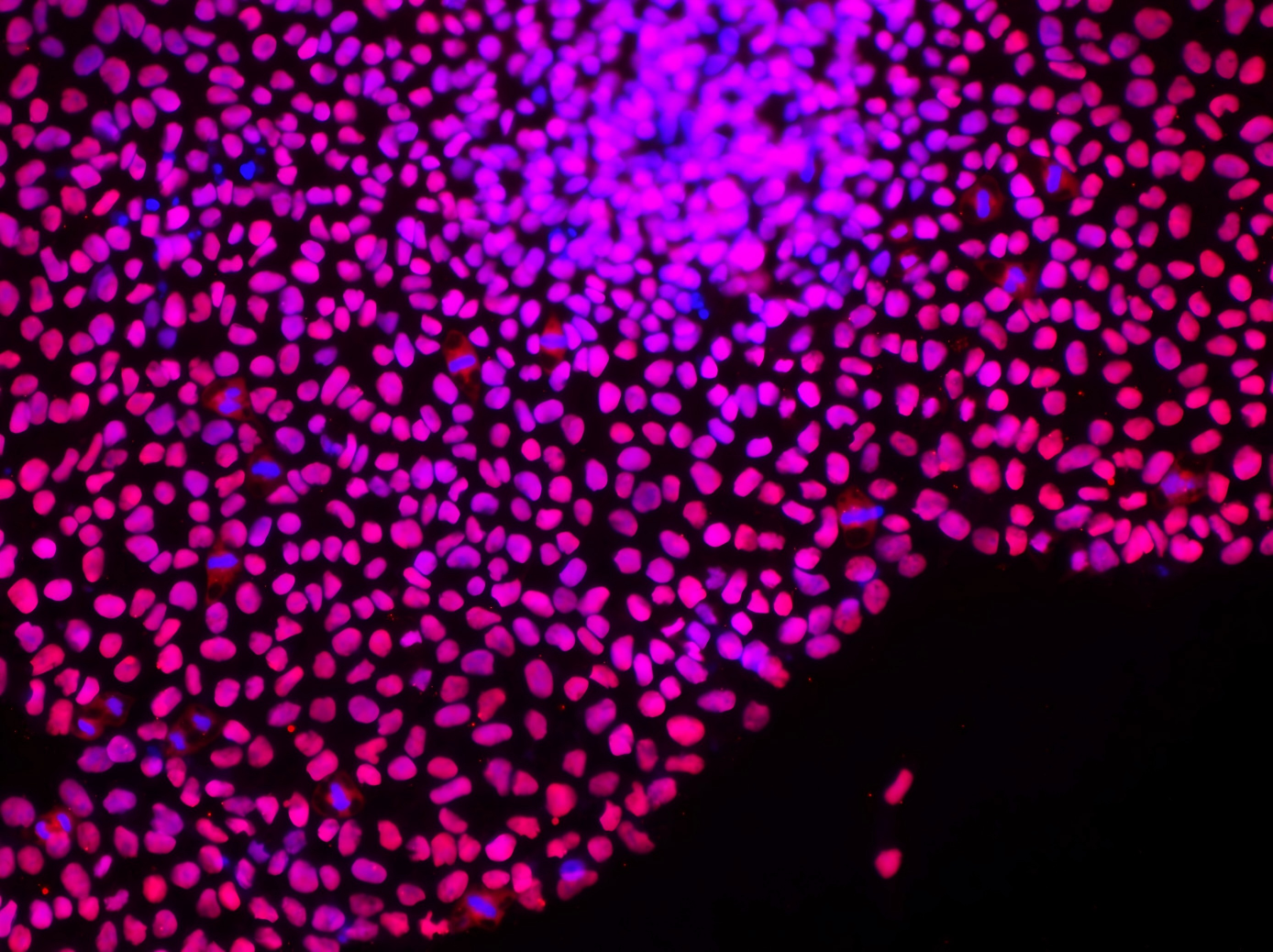

Supplement: Supplementary file 1 [file biology-15-00024-s001.zip › File S1. Figure1-original images/H9-OCT4/OCT4-DAPI.png]

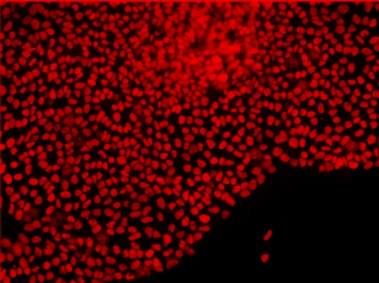

Supplement: Supplementary file 1 [file biology-15-00024-s001.zip › File S1. Figure1-original images/H9-OCT4/OCT4.png]

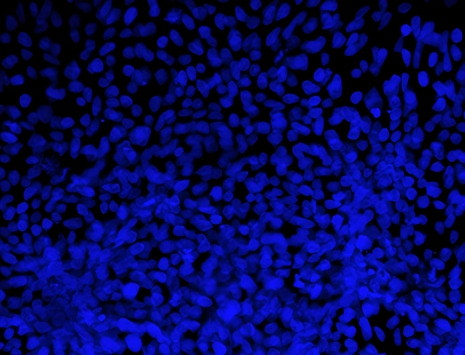

Supplement: Supplementary file 1 [file biology-15-00024-s001.zip › File S1. Figure1-original images/NPC/DAPI.png]

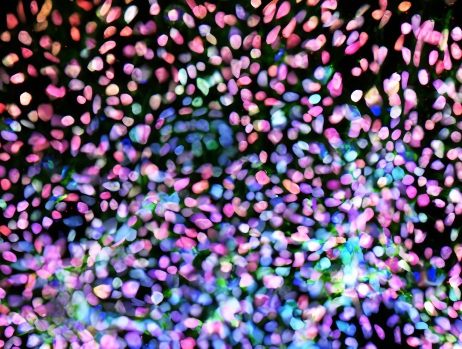

Supplement: Supplementary file 1 [file biology-15-00024-s001.zip › File S1. Figure1-original images/NPC/PAX6-SOX1-DAPI.png]

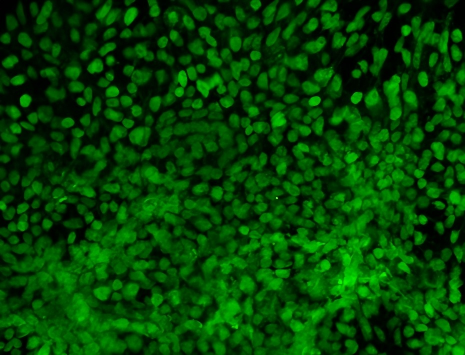

Supplement: Supplementary file 1 [file biology-15-00024-s001.zip › File S1. Figure1-original images/NPC/PAX6.png]

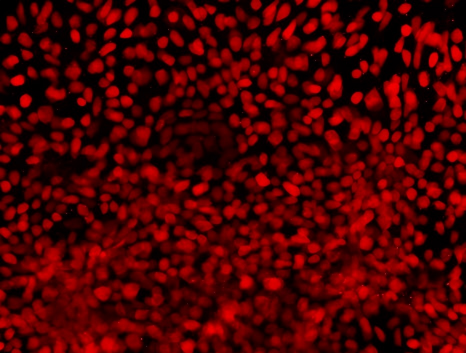

Supplement: Supplementary file 1 [file biology-15-00024-s001.zip › File S1. Figure1-original images/NPC/SOX1.png]

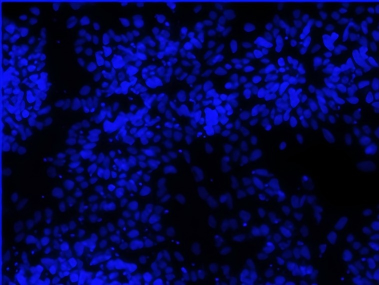

Supplement: Supplementary file 1 [file biology-15-00024-s001.zip › File S1. Figure1-original images/NSC/DAPI.png]

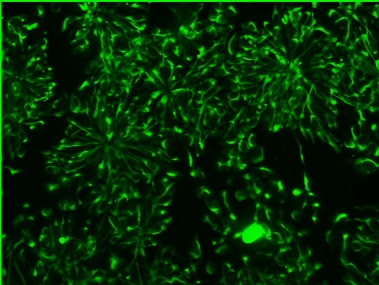

Supplement: Supplementary file 1 [file biology-15-00024-s001.zip › File S1. Figure1-original images/NSC/NESTIN.png]

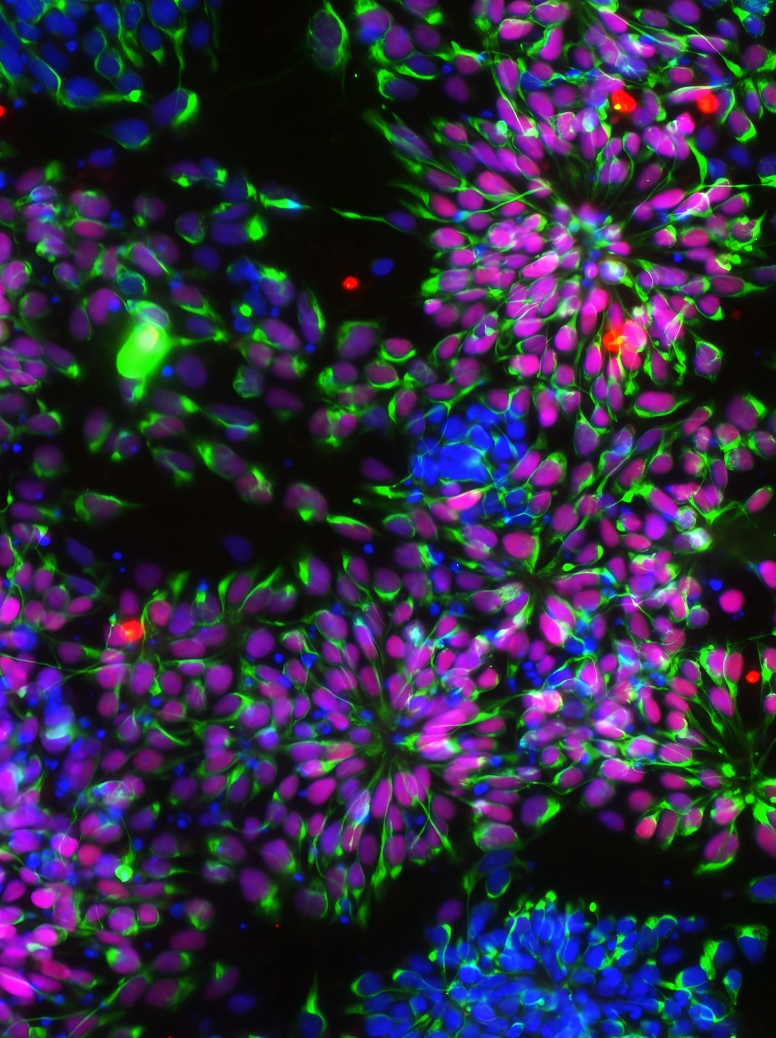

Supplement: Supplementary file 1 [file biology-15-00024-s001.zip › File S1. Figure1-original images/NSC/PAX6-NESTIN-DAPI.png]

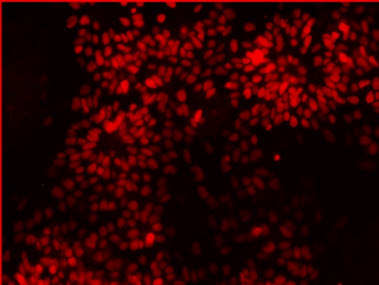

Supplement: Supplementary file 1 [file biology-15-00024-s001.zip › File S1. Figure1-original images/NSC/PAX6.png]
